# Clinical and enzymatic evaluation of the effect of dietary vitamin C on orthodontic tooth movement

**DOI:** 10.3389/fdmed.2026.1862437

**Published:** 2026-07-01

**Authors:** Naveen Pulikkottil Sunny, M. S. Ravi

**Affiliations:** Department of Orthodontics and Dentofacial Orthopaedics, AB Shetty Memorial Institute of Dental Sciences, Nitte (Deemed to be University), Mangalore, India

**Keywords:** accelerated orthodontics, canine retraction, gingival crevicular fluid (GCF), orthodontic tooth movement (OTM), periodontal ligament (PDL), vitamin C supplementation

## Abstract

**Aim and objectives:**

To evaluate the effect of dietary vitamin C supplementation on the rate of orthodontic tooth movement during canine retraction by comparing individuals receiving supplementation with those who are not. Additionally, to analyze changes in selected enzymatic biomarkers in the gingival crevicular fluid (GCF).

**Materials and methods:**

This study included 28 patients who underwent orthodontic treatment followed by first premolar extraction and levelling. Participants were divided into two groups: Group I received dietary vitamin C supplementation (500 mg chewable tablets), while Group II served as controls without supplementation. Canine retraction was carried out using NiTi closed coil springs delivering 150 g force with absolute anchorage provided by mini-implants, and digital scans were recorded at R0(before retraction) and R1(75 days). The rate of canine retraction was calculated as the distance moved per time. GCF was collected at baseline (T0), before retraction (T1), and after 75 days (T2) and analyzed for alkaline phosphatase and acid phosphatase levels using ELISA.

**Results:**

The vitamin C group demonstrated significantly greater maxillary canine retraction compared to controls, with mean values of (3.20 ± 0.84 mm) vs. (1.96 ± 0.60 mm) in males and (2.86 ± 0.38 mm) vs. (1.73 ± 0.31 mm) in females. Alkaline phosphatase and Acid phosphatase levels increased significantly over time in both groups, with a greater rise in the vitamin C group.

**Conclusion:**

Dietary vitamin C supplementation significantly accelerated canine retraction and enhanced bone remodeling enzyme activity during orthodontic treatment.

## Introduction

The prolonged duration of comprehensive orthodontic treatment has consistently been a major concern. In an effort to reduce overall treatment time with fixed appliances, several techniques have been developed to accelerate orthodontic tooth movement (OTM). The prolonged duration of Orthodontic treatment can lead to various detrimental effects, such as gingival inflammation (1), white spot lesions, dental caries, and root resorption (2), along with the possibility of reduced compliance towards the treatment. Due to these reasons, clinicians and researchers from all over the world have employed different techniques to enhance the rate of tooth movement.

Vitamin C, also known as Ascorbic acid, is a water-soluble vitamin having a crucial role in maintaining overall health and well-being due to its myriad of beneficial functions. Miresmaeili et al. observed that oral Vit C increases OTM in rats with more osteoclast lacunae around the root in the pressure area (3). Tankura et al. revealed the acceleratory effect of dose-dependent Vit C on OTM (4). Vit C has been reported to be involved in bone remodeling processes, which can accelerate tooth movement (5).

These procedures influence the cellular and microbiological environment and are associated with changes in the local biological response. Numerous biomarkers have been identified in gingival crevicular fluid (GCF) (6, 7).

Alkaline Phosphatase (ALP) and Acid Phosphatase (ACP) are associated with bone metabolism (8). ACP levels are associated with bone-resorbing cells such as osteoclasts and macrophages (9). On the opposite side, ALP levels are associated with bone-forming cells, mainly osteoblasts (10).

These proteins can be measured using an Enzyme-Linked Immunosorbent Assay (ELISA) kit. It has been found that the levels of ACP and ALP are increased during OTM as compared to normal values (11–16).

Studies have correlated the rate of tooth movement and root resorption with that of the enzymes and protein levels in GCF. With this literature background, the present study is designed and planned to clinically and enzymatically evaluate the effects of dietary vitamin C on orthodontic tooth movement in humans.

## Materials and methods

### Ethical considerations

Ethical approval for the study was obtained from the Institutional Ethics Committee prior to commencement of the study Ref. No. ETHICS/ABSMIDS/420/2024. Written informed consent was obtained from all participants after explaining the nature and objectives of the study. The study was conducted in accordance with the principles of the Declaration of Helsinki. This prospective clinical study included 28 individuals (18–24 years, mean age: 21 ± 3 years). The sample comprised 14 females and 14 males undergoing orthodontic treatment with first premolar extractions and maxillary canines retraction using NiTi closed coil springs. Participants were randomly allocated to the vitamin C supplementation group and control group using a computer-generated randomization sequence. Randomization was performed at the patient level since vitamin C was given orally and acts systemically. Hence, a split-mouth study could not be performed. Allocation concealment was done using sealed opaque envelopes.

Group I: Individuals taking dietary vitamin C supplements

Group II: Individuals without taking dietary vitamin C supplements

### Inclusion and exclusion criteria

Patients with minimal or no anterior crowding, healthy periodontal and gingival conditions, requiring orthodontic treatment with 0.022-inch MBT technique, bimaxillary protrusion, and high anchorage cases needing first premolar extraction and canine retraction as part of treatment, and providing informed consent were eligible. Exclusion criteria included medically compromised individuals and individuals with vitamin C deficiency. Individuals with severe skeletal malrelationships, poor hygiene, systemic conditions, craniofacial anomalies, or who were noncooperative were excluded.

### Sample size calculation

Sample size was calculated using N Master software with standard deviations of 0.05 and 0.06 and a mean difference of 0.03, with power = 95% and alpha value of 0.05. The minimum required sample size was 14 per group, giving a total of 28 participants. The study flow is presented using a CONSORT diagram, which is provided in the Supplementary Material.

### Methodology

Thorough clinical examination and medical histories were recorded. Patients meeting the inclusion and exclusion criteria underwent first premolar extraction before an initial leveling and alignment phase. 0.022″ slot MBT prescription stainless steel brackets(ORMCO, California, USA) were used to bond following standard protocols. Before retraction, baseline (R0) digital scans were taken using a 3Shape TRIOS 3 intraoral scanner (3Shape A/S, Copenhagen, Denmark). Segmental maxillary canine retraction was performed with NiTi closed coil springs (KODEN, KCK dental Pvt Ltd, Kerala, India) on 0.017 × 0.025 stainless steel arch wires, applying a standardized 150 g force per side measured with a Dontrix gauge. Absolute anchorage was maintained by using mini-implants. Individuals were supplied with vitamin C supplements (Limcee®, Abbott Healthcare Pvt. Ltd.) 500 mg in chewable tablets given by investigators, which were taken 1 tablet per day after dinner from the first day of retraction and continued till 75 days of retraction. The study was conducted over a 75-day observation period.

Throughout the orthodontic treatment, strict hygiene measures were consistently maintained to ensure optimal oral health. Follow-up phone calls were made to guide and support the patient in adhering to dietary restrictions and recommended the supplement intake. Clear instructions were provided to help maintain a proper diet and hygiene, including limiting the consumption of high-fiber foods, caffeine, calcium-rich items, and citrus or other acidic foods, as these may affect the treatment progress.

To evaluate the rate of canine retraction, digital impressions were obtained at two predetermined time points throughout the study.

1. At the start of retraction (R0)
2. Upon completion of the 75-day retraction (R1)

**Figure 1:**
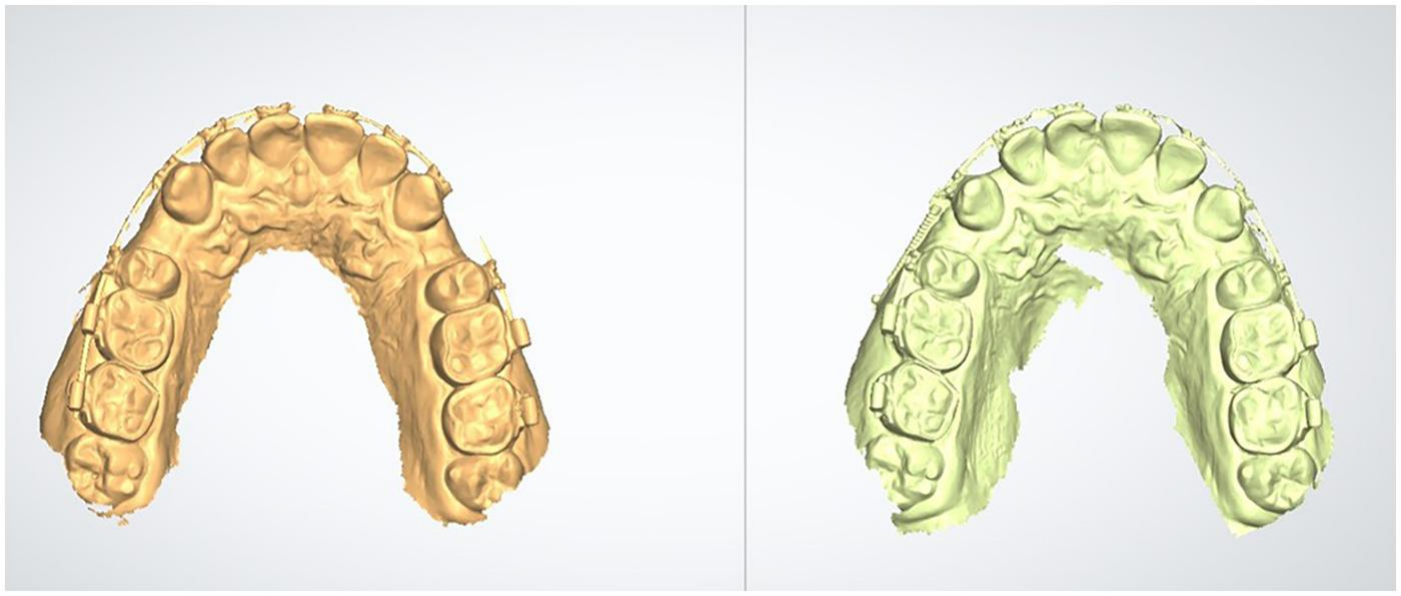
Intraoral scans of R0 and R1.

The scanned data was imported into the software in the form of a mesh format, which was STL mesh. After importing the scan data, it was oriented within a standardized coordinate system to obtain accurate values (Figure 1). The distance between the canine cusp tip and the mesiobuccal cusp of the first molar was measured bilaterally at each of these time points using a linear dimension measurement tool. R0 was designated as the reference scan, providing a baseline for comparison, and R1 was treated as measured data, representing progressive changes in tooth positioning. Alignment of Data: Provides alignment tools for accurate superimposition of scanned data in the same coordinate system. This step was critical to ensure that the comparison is meaningful and that both data sets are properly aligned for the analysis.

**Figure 2:**
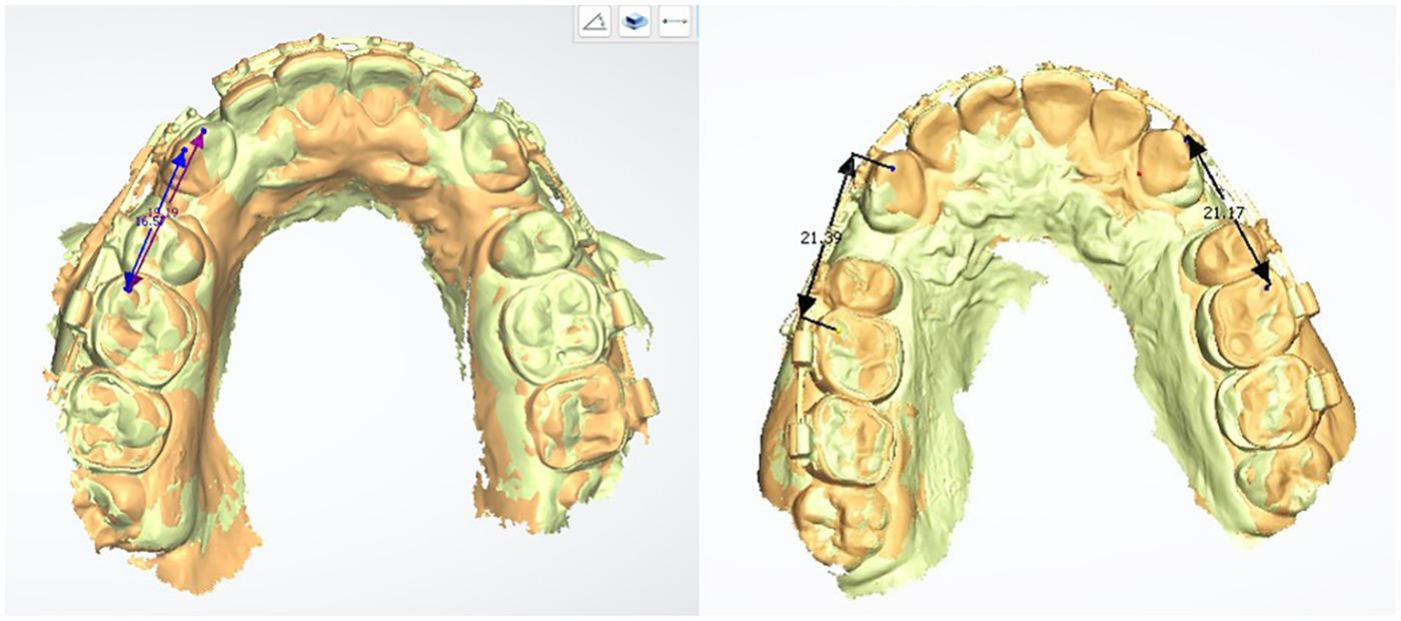
Scanned model with superimposition and measurements.

The overall movement throughout the study was determined by the difference in measurements between R0 and R1, which gave the distance of canine retraction. The rate of retraction was calculated by dividing the distance moved (in millimeters) by the corresponding time period. (Figure 2)

#### GCF sample collection

**Figure 3:**
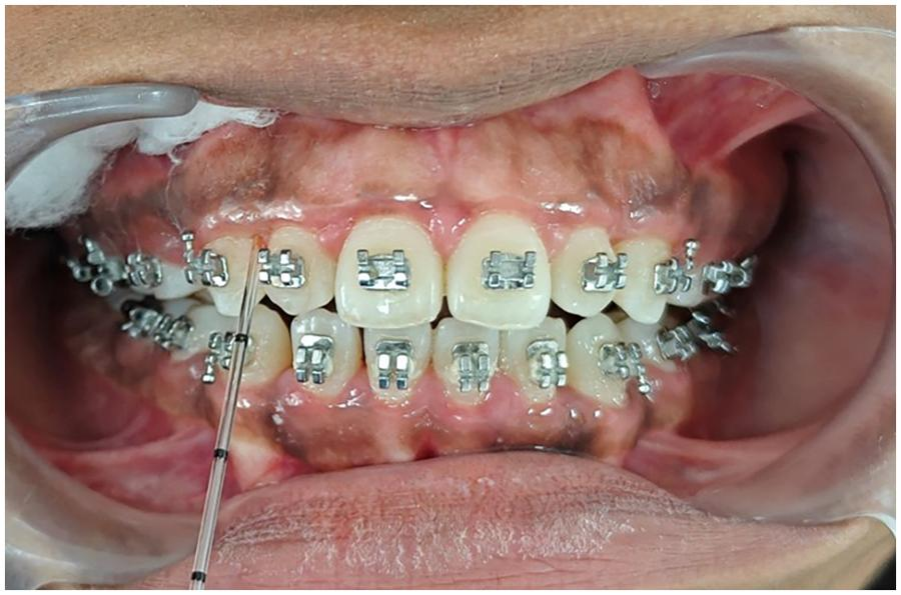
GCF collection.

GCF collection was standardized to minimize variability and ensure the reliability of biomarker measurements. Several factors, including the collection site, time of sampling, duration of collection, volume of GCF obtained, degree of gingival inflammation, contamination with saliva or blood, and mechanical stimulation of the gingival tissues, can significantly influence the concentration of biomarkers present in GCF. Therefore, all samples were collected using a uniform protocol, from the same predetermined sites and under identical clinical conditions and time intervals. The crevicular site of the gingiva was dried with an air syringe, and the teeth were isolated using a cotton roll. The sample site for the collection of GCF was standardized for all individuals to the mesiobuccal and distobuccal sites of the maxillary canines for ease of collection. 1–5 µ*L* calibrated micro capillary tube was used to collect 1µ*L* of the fluid by placing the volumetric micropipette extra crevicularly (Figure 3). The samples were collected at the following intervals

T0: before orthodontic treatment
T1: Prior to orthodontic retraction of the canine
T2: 75 days after commencement of retraction of the canine

GCF samples were collected and evaluated for the levels of Alkaline Phosphatase and Acid Phosphatase using a commercially available enzyme-linked immunosorbent assay (ELISA) kit at these intervals.

#### Sample preparation

The GCF samples were transferred to an Eppendorf tube containing 100 µ*L* phosphatase-buffered saline of pH 7, which was transferred to a vial using a 100 µ*L* micropipette.

The vials containing GCF samples were sealed, labelled, and immediately transferred to the laboratory, where it was centrifuged for 1 min for removal of the bacterial and cellular debris.

Following centrifugation, the supernatant was collected and stored in a laboratory deep freezer at −80 °C until further analysis.

#### Elisa preparation

The samples were analysed for Acid phosphatase and Akaline phosphatase using a commercially available ELISA kit (*REED BIOTECH ELISA*). ELISAs were usually carried out using 96-well polystyrene plates that allow passive binding of antibodies and proteins. This efficient binding and immobilization of reagents is what makes ELISA techniques simple to design and easy to perform.

This assay followed the sandwich ELISA method. Pre-coated microwells were used to bind ACP and ALP from samples, followed by incubation with biotinylated detection antibodies, enzyme conjugates, and chromogenic substrate. The intensity of color developed, corresponding to the amount of ACP and ALP, was measured using a microplate reader at 450 nm. The concentrations of human ALP and ACP in the samples were subsequently calculated by correlating the optical density (OD) values with the corresponding standard curve.

### Statistical analysis

A total of 40 participants were initially enrolled; 12 were lost during follow-up, resulting in a final sample of 28 participants for analysis. All data were entered in Excel (Microsoft, Redmond, WA, USA) and analyzed using IBM SPSS Statistics (Version 23, IBM, Armonk, NY, USA). The Shapiro–Wilk test was used to assess the normality of the variables. Since the variables were normally distributed, parametric tests were applied to the data. Descriptive statistics were expressed as mean and standard deviation. The effects of dietary Vitamin C supplementation and gender on the rate of canine retraction and enzyme levels were analyzed using two-way repeated-measures ANOVA, followed by *post hoc* pairwise comparisons with Bonferroni correction to identify specific differences between time points. A *p*-value < 0.05 was considered statistically significant.

## Results

### Rate of canine retraction

Participants in the Vit C group demonstrated higher mean canine retraction compared to those in the non–Vit C group in both males and females. Among males, the mean canine retraction was 3.20 ± 0.84 mm in the Vit C group and 1.96 ± 0.60 mm in the non–Vit C group, while among females it was 2.86 ± 0.38 mm and 1.73 ± 0.31 mm, respectively.

Two-way analysis of variance was performed to evaluate the effects of vitamin C supplementation and gender on canine retraction. The analysis showed a statistically significant main effect of group (F = 30.13, *p* = 0.000), partial *η*² = 0.557, showing a large effect, indicating that Vit C supplementation had a significant influence on the rate of canine retraction. The main effect of gender was not statistically significant (F = 1.75, *p* = 0.199) and partial *η*² = 0.068, suggesting that canine retraction did not differ significantly between males and females when adjusted for group. The interaction between group and gender was also not statistically significant (F = 0.06, *p* = 0.814), indicating that the effect of Vit C on canine retraction was comparable in both males and females. The overall model accounted for 57.1% of the variance in canine retraction (R² = 0.571). (Table 1)

**Table 1:** Comparison of the rate of canine retraction in group I and group II.

Dependent variable: canine retraction				
Group	Gender	Mean(mm)	Std. deviation	N
With vitamin C	Male	3.1986	.83878	7
	Female	2.8614	.38253	7
	M + F	3.0300	.65027	14
Without vitamin C	Male	1.9600	.60415	7
	Female	1.7257	.30812	7
	M + F	1.8429	.47650	14

### Comparison of alkaline phosphatase

The mean ALP levels at baseline (T0) were 603.03 ± 151.15 pg/mL in the Vit C group and 545.91 ± 102.51 pg/mL in the non-Vit C group, indicating comparable starting levels. At T1, ALP increased to 1,056.21 ± 129.43 pg/mL in the Vit C group and 863.11 ± 82.32 pg/mL in the non-Vit C group. By T2, the levels further increased to 1,660.91 ± 81.40 pg/mL in the Vit C group and 1,307.51 ± 102.60 pg/mL in the non-Vit C group, indicating that ALP levels increased over time in both groups, with a significantly greater rise observed in individuals receiving Vit C supplementation. (Table 2).

**Table 2:** Comparison of alkaline phosphatase in group I and group II across different time intervals.

Time	N	ALP with vitamin C		ALP without vitamin C	
		Mean(pg/mL)	Std. deviation	Mean(pg/mL)	Std. Deviation
T0	14	603.029	151.1493	545.907	102.5068
T1	14	1,056.214	129.4297	863.107	82.3168
T2	14	1,660.914	81.4003	1,307.514	102.5968

### Comparison of acid phosphatase

The mean ACP levels at baseline (T0) were 0.726 ± 0.049 ng/mL in the Vit C group and 0.634 ± 0.188 ng/mL in the control group, indicating comparable starting levels. At T1, ACP increased to 0.819 ± 0.047 ng/mL in the Vit C group and 0.774 ± 0.044 ng/mL in the control group. By T2, the levels further increased to 0.861 ± 0.251 ng/mL in the Vit C group and 0.837 ± 0.020 ng/mL in the non-Vit C group. Although the main effects of time (F = 1.404, *p* = 0.264) and group (F = 0.662, *p* = 0.431) were not statistically significant, the time × group interaction was significant (F = 11.756, *p* = 0.000), indicating that ACP levels changed differently over time in the two groups, with the Vit C group showing a slightly higher trajectory compared to controls. (Table 3)

**Table 3:** Comparison of acid phosphatase in group I and group II in different time intervals.

Time	N	ACP with vitamin C		ACP without vitamin C	
		Mean(ng/mL)	Std. deviation	Mean(ng/mL)	Std. deviation
T0	14	0.726	0.049	0.634	0.188
T1	14	0.819	0.047	0.774	0.044
T2	14	0.861	0.251	0.837	0.020

## Discussion

There is a noticeable lack of clinical studies evaluating the role of dietary Vit C supplementation in accelerating OTM in humans, which limits definitive conclusions regarding its clinical effectiveness. Owing to this gap in the literature, understanding the extent to which Vit C can influence the rate of tooth movement remains challenging. Gaining insight into its efficiency is essential for refining treatment protocols and identifying simple adjunctive measures that can enhance orthodontic outcomes. The effect of oral vitamin C was not previously reported on orthodontic tooth movement in humans; the present study has shown that oral vitamin C has a significant role in orthodontic tooth movement, demanding further longitudinal studies. Within limitations, oral vitamin C supplementation was effective. Canine retraction was chosen as the focus of evaluation in the present study because it represents one of the most time-consuming phases of orthodontic space closure. Any intervention capable of accelerating this stage has the potential to significantly reduce overall treatment duration and improve patient compliance and satisfaction. While Vit C accelerates tooth movement by enhancing cellular and enzymatic activity within the periodontal ligament and alveolar bone, it does not alter the biological limits of orthodontic treatment. Unlike corticotomy-based techniques that may modify alveolar bone architecture or increase periodontal support, Vit C supplementation primarily influences the rate of bone turnover without structural modification. It is a key cofactor for prolyl and lysyl hydroxylase enzymes, which are essential for collagen maturation and stabilization within the periodontal ligament and alveolar bone, allowing these tissues to withstand and adapt to orthodontic forces. Vit C also enhances osteoblastic differentiation and activity, increasing alkaline phosphatase expression and promoting new bone formation on the tension side of the moving tooth. Simultaneously, through its antioxidant and anti-inflammatory actions, vitamin C regulates cytokine release and reduces oxidative stress, thereby modulating osteoclastic activity on the pressure side and supporting a controlled, efficient, and biologically favorable orthodontic tooth movement process. The tolerable upper intake level for vitamin C has been established at 2000 mg (2 g) per day (18). Very high intakes may occasionally cause mild gastrointestinal disturbances such as nausea, diarrhea, or abdominal cramps, but serious adverse effects are uncommon. Several studies and reviews have reported that vitamin C is generally safe even at higher doses, with toxicity observed only at extremely excessive intakes over prolonged periods (18).

This study contributes valuable clinical insight into the effectiveness of Vit C supplementation in accelerating canine retraction and its potential unintended effects on anchorage units, such as molar movement. By evaluating both desired and collateral tooth movement, the findings help determine the clinical relevance of Vit C as an adjunctive aid in orthodontics. Overall, Vit C supplementation appears to be a practical, patient-friendly option that enhances treatment efficiency while maintaining biological safety, making it a promising aid for routine orthodontic care. Canine retraction for space closure was carried out using NiTi closed coil springs, which are known for delivering a relatively constant force within the manufacturer's recommended activation range. The initial force was standardized at 150 grams to achieve controlled and efficient tooth movement. To eliminate unwanted anchorage loss, absolute anchorage was employed using mini implants, ensuring stability of the posterior teeth and allowing canine retraction to proceed without molar movement during the space closure phase. The 75-day observation period was selected because orthodontic tooth movement is a biological process that requires sufficient time for periodontal ligament remodeling, osteoclastic bone resorption, and osteoblastic bone formation to produce clinically measurable tooth movement. The initial displacement and lag phases are followed by an acceleration and linear phase, during which most clinically significant tooth movement occurs as a result of active alveolar bone remodeling. Therefore, an observation period of approximately 10–12 weeks (75 days) was considered appropriate to evaluate the effect of the intervention on orthodontic tooth movement. Previous evidence indicates that bone remodeling and consolidation associated with orthodontic tooth movement are typically assessed over periods ranging from 8 to 12 weeks. Verdecchia et al. reported that several authors recommend allowing 8–12 weeks for bone consolidation and remodeling before evaluating orthodontic movement outcomes (19). Furthermore, Li et al. emphasized that orthodontic tooth movement is mediated by sequential biomechanical and biological responses involving periodontal ligament and alveolar bone remodeling, processes that require several weeks to become clinically evident (20). Accordingly, a 75-day interval was chosen as a biologically relevant and clinically meaningful timeframe to detect measurable differences in tooth movement while ensuring adequate tissue response. The rate of canine retraction was evaluated using digital impressions made at two defined time intervals: at initiation of retraction (R0), and after completion of the 75-day retraction period (R1). At all time intervals, space closure was significantly higher in the vit C group compared to the non-vit C group. The main effect of gender was not statistically significant, suggesting that canine retraction did not differ significantly between males and females when adjusted for group. This means that vit C accelerated canine retraction by approximately 57.1% faster compared to non-vit C. Tanaka indicated that there is a notable change between the control and 1,000 mg/day vit C groups at day 14, suggesting that a 1,000 mg/day vit C supplement could enhance tooth movement without clear effects on osteoclast profile and inter- radicular bone areas (4). Bolat investigated the effects of vitamins C and E on experimental OTM and showed a significant increase in osteoblastic activity and positively influenced collagen fiber numbers and bone formation in the tension side compared to the control group (17).

Gingival crevicular fluid (GCF) contains numerous measurable biomarkers that help assess the health and status of the periodontium (6). These biomarkers include ALP, an indicator of osteoblastic activity and bone formation, and ACP reflecting osteoclastic activity and is associated with bone resorption. Since the levels of these enzymes are known to vary during puberty, the study sample was limited to individuals aged 18–24 years. Male and female participants were not analyzed separately, as previous reports indicate that enzyme levels do not show significant differences between genders. GCF samples were collected using a calibrated micropipette at T_O_ (before orthodontic treatment) and at time intervals T1 (before starting retraction) and T2 (on the 75th day of retraction). These samples were analysed for Acid phosphatase and Akaline phosphatase levels using a commercially available ELISA kit.

There was a significant rise in acid phosphatase levels in the Vitamin C group during the 75 days, suggesting increased osteoclastic activity and bone turnover, which has facilitated a higher rate of orthodontic tooth movement. Alkaline phosphatase levels increased progressively from T0 to T2 in the Vitamin C group, indicating increased osteoblastic activity and bone formation during orthodontic tooth movement. Future studies incorporating more frequent and standardized sampling intervals, along with activity-based enzyme assays, are recommended to provide a more comprehensive understanding of the biochemical changes associated with orthodontic tooth movement.

### Limitations of the study

One of the limitations of the present study is the method used for biochemical analysis. Enzyme levels in the GCF were assessed using ELISA, which primarily quantifies the concentration of enzymes rather than their functional activity. Since enzyme activity provides a more accurate reflection of ongoing biological processes, the use of specific enzyme activity assays could have yielded more precise information regarding osteoblastic, osteoclastic, and inflammatory activity during orthodontic tooth movement. Another limitation relates to the timing of GCF sample collection. The sampling intervals in this study may not have fully captured the dynamic changes in enzyme activity that occur during the initial phase of orthodontic force application.

## Conclusion

Within the limitations of the study, it may be concluded that Individuals receiving dietary Vit C supplementation demonstrated a significantly greater rate of maxillary canine retraction compared to those who did not receive supplementation, indicating a positive influence of Vit C on OTM. The effect of Vit C on canine retraction was comparable in both males and females, as no statistically significant interaction was observed. Alkaline phosphatase and acid phosphatase levels in GCF showed a greater significance in the Vit C group, indicating enhanced osteoblastic activity and bone formation associated with Vit C supplementation during OTM.

Overall, the findings of the present study suggest that dietary Vit C supplementation enhances the biological responses associated with orthodontic tooth movement by influencing bone remodeling enzymes, which is reflected in accelerated canine retraction.

## Data Availability

The original contributions presented in the study are included in the article/Supplementary Material, further inquiries can be directed to the corresponding author.
