# Supplementary material for: Clinical and enzymatic evaluation of the effect of dietary vitamin C on orthodontic tooth movement
Source: Front Dent Med. 2026 Jul 1;7:1862437. doi: 10.3389/fdmed.2026.1862437 (PMC13370340; doi:10.3389/fdmed.2026.1862437)
Supplement: Supplementary file 1 [file Supplementaryfile1.doc]

**CONSORT 2010 Flow Diagram**

**Allocation**

**Analysis**

**Follow-Up**

**Enrollment**

Assessed for eligibility (n=28)

Excluded (n=0)

♦  Not meeting inclusion criteria (n=0)

♦  Declined to participate (n=0)

♦  Other reasons (n=0)

Analysed (n=14)
♦ Excluded from analysis (give reasons) (n=0)

Lost to follow-up (give reasons) (n=0)

Discontinued intervention (give reasons) (n=0)

Allocated to Vitamin C supplementation (n=14)

♦ Received allocated intervention (n=14)

♦ Did not receive allocated intervention (give reasons) (n=0)

Lost to follow-up (give reasons) (n=0)

Discontinued intervention (give reasons) (n=0)

Allocated to Control group (n=14)

♦ Received allocated intervention (n=14)

♦ Did not receive allocated intervention (give reasons) (n=0)

Analysed (n=14)
♦ Excluded from analysis (give reasons) (n=0)

Randomized (n=28)
